# Decoupling Analysis of Water Footprint and Economic Growth: A Case Study of Beijing–Tianjin–Hebei Region from 2004 to 2017

**DOI:** 10.3390/ijerph16234873

**Published:** 2019-12-03

**Authors:** Yang Kong, Weijun He, Liang Yuan, Juqin Shen, Min An, Dagmawi Mulugeta Degefu, Xin Gao, Zhaofang Zhang, Fuhua Sun, Zhongchi Wan

**Affiliations:** 1 Business School, Hohai University, Nanjing 211100, China; yangkong@hhu.edu.cn (Y.K.); jqshen@hhu.edu.cn (J.S.); gxtz1987@hhu.edu.cn (X.G.); zackzhang@hhu.edu.cn (Z.Z.); fhsun@hhu.edu.cn (F.S.); 2 College of Economics and Management, China Three Gorges University, Yichang 443002, China; anmin@hhu.edu.cn (M.A.); dagmawi.degefu@ryerson.ca (D.M.D.); zhongchi_wan@ctgu.edu.cn (Z.W.); 3 College of Agricultural Engineering, Hohai University, Nanjing 211100, China; 4 Faculty of Engineering and Architectural Science, Ryerson University, Toronto, ON M5B 2K3, Canada

**Keywords:** Beijing–Tianjin–Hebei region, water footprint, economic growth, coordinated development, decoupling

## Abstract

The Beijing–Tianji–Hebei region (BTHR) is economically developed and densely populated, but its water resources are extremely scarce. A clear understanding of the decoupling relationship between water footprint and economic growth is conducive to facilitating and realizing the coordinated development of water resources and economic growth in this region. This study calculated the water footprint and other related indicators of BTHR from 2004 to 2017, and objectively evaluated the utilization of water resources in the region. Then, logarithmic mean divisia index (LMDI) method was applied to study the driving factors that resulted in the change of water footprint and their respective effects. Finally, Tapio decoupling model was used to research the decoupling relationships between water footprint and economic growth, and between the driving factors of water footprint and economic growth. There are three main results in this research. (1) The water utilization efficiency in BTHR continues to improve, and the water footprint shows a gradually increasing trend during the research period, among which the agricultural water footprint accounts for a relatively high proportion. (2) The change of water footprint can be attributed to efficiency effect, economic effect, and population effect. Furthermore, efficiency effect is the decisive factor of water footprint reduction and economic effect is the main factor of water footprint increase, while population effect plays a weak role in promoting the increase in water footprint. (3) The decoupling status between water footprint and economic growth show a weak decoupling in most years, while the status between water footprint intensity and economic growth always remains strong decoupling. Moreover, population size and economic growth always show an expansive coupling state. In sum, it is advisable for policy makers to improve water utilization efficiency, especially agricultural irrigation efficiency, to raise residents’ awareness of water conservation, and increase the import of water-intensive products, so as to alleviate water shortage and realize the coordinated development of water resources and economic growth in BTHR.

## 1. Introduction

Water is the most important renewable natural capital that nourishes life [1]. It is also crucial for sustainable economic development and environmental protection. With the remarkable growth of population and the rapid development of socio-economic sectors, demand for water is increasing sharply. However, due to the uneven spatial and temporal distribution, the limited water resources cannot meet the growing water demand [2], making about four billion people faced with varying degrees of water scarcity [3]. Thus, the demand–supply imbalance of water resources has become a crucial factor hindering the sustainable development of many countries [4].

As the world’s second-largest economy and the most populous country, China accounts for about 6% of the world’s total water resources, ranking fourth in the world. However, China’s per capita water resources rank 121st in the world, only a quarter of the world’s average level, making China one of the thirteen water-poor countries across the globe [5]. With the development and utilization of water resources and the aggravation of water pollution, China is facing an increasingly severe water crisis now [6]. Thus, how to effectively develop and utilize water resources to reduce the consumption of water resources per unit of economic growth has become a major concern in China.

The concept of water footprint was introduced in 2002 by Hoekstra [7]. It objectively reflects the actual water consumption by comprehensively measuring the freshwater consumption taking into account both direct and indirect water use. In recent years, studies on water footprint have increased dramatically. Most of the previous studies are rooted in the sustainability of water resources, focusing on water consumption calculation and analysis of water use efficiency [8,9,10]. As for research methods, the water footprint method [11,12,13,14] and the data envelopment analysis (DEA) model [15,16,17] are the ones that are mainly used. Though these methods can reflect the actual water consumption and water use efficiency, they fail to identify the driving factors of water consumption.

Since decomposition results are easy to be interpreted with the characteristics of uniqueness and consistency, the logarithmic mean divisia index (LMDI) method is widely used in the field of energy and environment [18,19,20], and gradually, its application to analyze the driving factors of water resources grew [21,22]. For instance, Li et al. (2018) [23] and Chen et al. (2017) [24] studied the changes of industrial water consumption and industrial wastewater discharge, respectively, and effectively identified the main influencing factors. Since economic growth is closely related to water resources consumption, it is necessary to explore their mutual dynamics. Though the LMDI method can identify the driving factors affecting the changes in water resources, it cannot quantitatively measure the decoupling status between economic growth and water consumption. Thus, decoupling analysis has been widely recognized for objectively measuring the relationship between environmental pressure and economic growth in recent years [25,26,27]. In 2005, Tapio firstly introduced decoupling elasticity into a decoupling model by studying the decoupling relationship between the development of the European transport industry and economic growth from 1997 to 2001 [28]. Since then, scholars have begun to study the decoupling relationship between water resources and economic growth using the Tapio decoupling model [29,30,31,32] and the results displayed different degrees of decoupling between water resources utilization and economic growth.

With the widespread application of LMDI method and Tapio decoupling model in the field of resources and environment, some studies on energy and carbon emissions combined with both methods have emerged [33,34,35]. Obviously, previous studies have provided a feasible way to research water resources. In order to realize the coordinated development between water consumption and economic growth, further studies should be conducted to identify the driving factors affecting water consumption, to explore the decoupling relationship between water consumption and economic growth, and to further analyze the decoupling states between the driving factors of water consumption and economic growth. Otherwise, policy makers cannot get targeted implications to promote the sustainable development of economy and water resources. Thus, this study explored the decoupling relationship between water resources consumption and economic growth, and between the influencing factors of water resources consumption and economic growth on the basis of the water footprint theory and the above two methods. Our research used the BTHR as a case study. With dense population and severe water scarcity, BTHR’s economic development is inevitably faced with critical water crisis. By studying the internal relationship between water resources and economic growth, policy makers in BTHR will be able to act accordingly with the help of the research results. 

As discussed above, there are three main contributions to this research. (1) In accordance with the water footprint theory, the actual water consumption in BTHR from 2004 to 2017 was accurately calculated and the utilization of water resources was effectively evaluated. (2) The driving factors affecting the change of water footprint and their respective effects were analyzed with LMDI method. (3) The decoupling status between water footprint and economic growth was explored. Furthermore, the decoupling states between the driving factors of water footprint and economic growth were explored.

The rest of this research is organized as follows: Section 2 introduces the data source, study area, and methodology. Section 3 shows the main results and discussion. The conclusions and policy implications are shown in Section 4.

## 2. Data Source and Methodology

### 2.1. Study Area

Beijing, Tianjin, and Hebei, closely linked geographically, not only have the same resource endowments and similar climate characteristics, but also share an integrated water resources system. With approximately 7.7% of China’s population, the Beijing–Tianjin–Hebei region (BTHR) covers 2.35% of the total land area of China and accounts for 9.32% of China’s gross domestic product (GDP). In addition, due to the implementation of the Belt and Road Initiative and the Beijing–Tianjin–Hebei Integration Strategy, the BTHR is officially recognized as a more important strategic development region of China, and its coordinated development can serve as a demonstration for the whole country.

**Figure 1:**
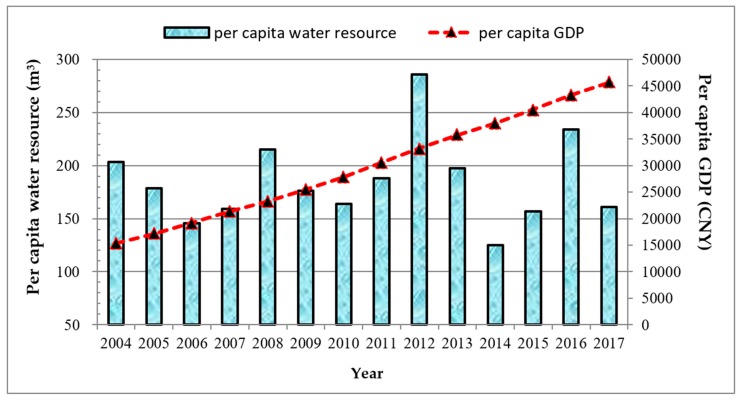
The change of per capita water resource and per capita gross domestic product (GDP) in Beijing–Tianjin–Hebei region (BTHR) (2004–2017).

**Figure 2:**
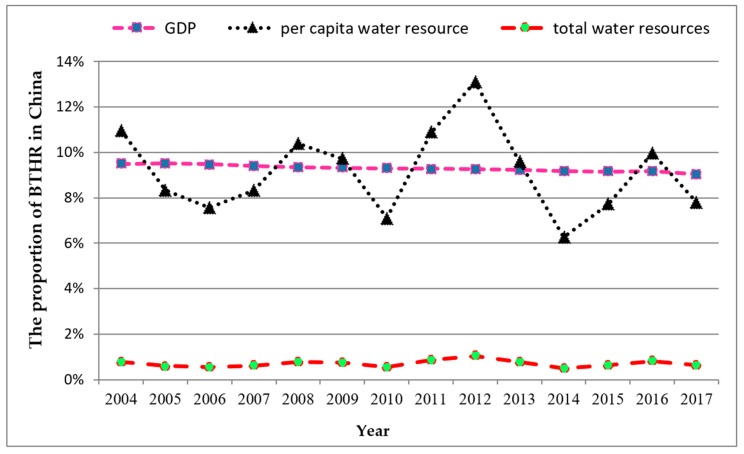
The proportion of Beijing–Tianjin–Hebei region (BTHR)’s gross domestic product (GDP), per capita water resource, and total water resources in China (2004–2017).

In order to simplify the description and facilitate the understanding of essential ideas, the changing trend of water resources and economic indicators in BTHR from 2004 to 2017 are shown in Figure 1 and Figure 2. As shown in the two figures, the mean annual per capita water resources in BTHR fluctuate around 186.78 (m^3^/(person·year)), accounting for less than 10% of the national average. Meanwhile, the annual discharge of industrial wastewater in BTHR is more than 1.2 billion tons from 2004 to 2015. The annual proportions of water reuse in Hebei and Tianjin are more than 90% during 2008 to 2015, while that of Beijing is less than 35%. Thus, it reflects the severe water crisis facing the BTHR [36,37]. Nevertheless, the per capita GDP in BTHR increased from 15,333 (CNY/person) in 2004 to 45,615 (CNY/person) in 2017. 

Despite its soaring economic growth, BTHR accounts for only 0.7% of China’s water resources. The shortage of water resources has inevitably become one of the critical factors restricting the sustainable development of the region’s socio-economic sectors. To sum up, on the basis of dynamic assessment of water resources utilization in BTHR, it is necessary to precisely identify the key indicators affecting water resource consumption and study the decoupling state between water footprint and economic growth, aiming to provide some effective and feasible implications for relevant policy makers.

### 2.2. Data Source

In addition to the water footprint and total water resources in Beijing, Tianjin, and Hebei from 2004 to 2017, this paper also collected the data of economic indicators such as GDP and import and export volume. The economic data of Beijing, Tianjin, and Hebei were obtained from the China Statistical Yearbook (2005–2018) [36], and the relevant indicators of water footprint were mostly from China Water Resources Bulletin (2004–2017) [37]. Agricultural products are divided into crops and livestock products. The agricultural water footprint is multiplied by the output of agricultural products and the virtual water content per unit of agricultural product [38,39]. Considering the complexity of the calculation process and the difficulty of obtaining relevant data, this study takes cotton, oil, fruit, grain, vegetables, and livestock products into account, whose specific reference values are shown in Table 1 [31,40]. The industrial water footprint, residential water footprint, and ecological environmental water footprint are represented as industrial water consumption, household water consumption, and ecological environment water consumption, respectively, whose values are directly derived from the water resources bulletin of corresponding years [31,41]. It is worth noting that the virtual water import and export was calculated by multiplying the total amount of import and export trade times the water consumption per 10,000 CNY of GDP [41,42]. Hence, the total amount of import and export trade should be converted from US dollars into CNY for better measurement because other economic indicators are expressed in CNY. Moreover, the authors assumed that 60% of the total water resources are dedicated to ecosystem protection, hence the available water resources (WA) equals the remaining amount [43,44]. To eliminate the impact of inflation, all the annual GDP indicators were converted into the real GDP at constant prices of 2000.

### 2.3. Water Footprint Method

Virtual water refers to the amount of water needed to produce products and services; it takes neither a certain period nor an area into account [45]. Based on the concept of virtual water, Hoekstra proposed the water footprint in 2002, which was defined as the total amount of water resources required by all the products and services consumed by a country, a region, or a person within a certain period of time [7]. Equations (1)–(3) below describe the concept.

(1) WF=IWF+EWF

(2) $$IWF=AWF+WF_{Industrial}+RWF+EEWF-VWE_{dom}$$

(3) $$EWF=VWI-VWE_{re-export}$$

In Equation (1), *WF* (water footprint) is the regional water footprint; *IWF* (internal water footprint of consumption) refers to the total amount of water resources consumed by the products and services consumed by residents in the region. *EWF* (external water footprint of consumption) denotes the imported virtual water consumed by local residents. In Equation (2), *AWF* (agricultural water footprint) represents the water consumption for agricultural production, including the water consumption for crops and animal products. $WF_{Industrial}$ (industrial water footprint) refers to the water consumption for industrial production; *RWF* (residential water footprint) denotes the water consumption of local residents; *EEWF* (ecological environmental water footprint) refers to the local ecological water consumption, which represents the water resources used for ecological environmental protection. It only includes environmental water supplied by human measures and water replenishment of some rivers, lakes, and wetlands, but does not include water content naturally satisfied by precipitation and runoff [31,37,40]. $VWE_{dom}$ (virtual water export) represents the virtual water quantity for export. In Equation (3), *VWI* (virtual water import) represents the total amount of virtual water imported from abroad; $VWE_{re-export}$ is the total amount of virtual water imported for re-export.

Based on previous literature [31,46,47,48], the specific meanings and relevant calculation formulas of per capita water footprint, water import dependency, water self-sufficiency, water scarcity, and water footprint intensity are given in Table 2.

(4) $$PWF=\frac{WF}{P}$$

(5) $$WD=\frac{EWF}{WF}\times 100\%$$

(6) $$WSS=\frac{IWF}{WF}\times 100\%$$

(7) $$WS=\frac{WF}{WA}\times 100\%$$

(8) $$WFI=\frac{WF}{GDP}$$

### 2.4. LMDI Model

This study applied LMDI model to decompose the influencing factors of water footprint into water footprint intensity, economic level, and population size [49]. The specific formula is shown below:

(9) $$WF_{t}=\sum_{i}WF_{i,t}=\sum_{i}\frac{WF_{i,t}}{GDP_{i,t}}\cdot \frac{GDP_{i,t}}{P_{i,t}}\cdot P_{i,t}=\sum_{i}EFF_{i,t}\cdot ECO_{i,t}\cdot P_{i,t}.$$

In Equation (9), ${\mathrm{WFP}}_{t}$ represents the water footprint of BTHR in the year of *t*. $WF_{i,t},GDP_{i,t}$, and $P_{i,t}$ are respectively the water footprint, actual GDP, and year-end resident population of *i* province or municipality in the year of *t*. Additionally, $EFF_{i,t}$ refers to the water footprint intensity (*WFI*), indicating the amount of water footprint required to produce per unit of GDP. The larger this index is, the lower the water utilization efficiency is. $ECO_{i,t}$ denotes the economic level (*EL*), representing the per capita GDP. The larger the index is, the greater the impact of economic development level on water footprint is. Moreover, $P_{i,t}$ is the population size (*PS*), represented by the resident population at the end of the year. Therefore, the total effect (∆W) of water footprint, calculated by the sum of three decomposition effects, represents the total change of water footprint [23]. Where efficiency effect is ∆EFF, the economic effect is denoted as ∆ECO and population effect is represented as ∆P. Furthermore, the calculation formulas for each decomposition effect are shown in Equations (11)–(13). In this research, each decomposition effect represents the change of water footprint caused by this effect when the other two decomposition effects remain unchanged. If the value of decomposition effect is positive, it indicates that the driving factor promotes the increase in water footprint, and vice versa.

(10) ∆W=∆EFF+∆ECO+∆P

(11) $$∆EFF=\underset{i}{\sum }\frac{WFP_{i,t}-WFP_{i,0}}{lnWFP_{i,t}-lnWFP_{i,0}}ln\frac{EFF_{i,t}}{EFF_{i,0}}$$

(12) $$∆ECO=\underset{i}{\sum }\frac{WFP_{i,t}-WFP_{i,0}}{lnWFP_{i,t}-lnWFP_{i,0}}ln\frac{ECO_{i,t}}{ECO_{i,0}}$$

(13) $$∆P=\underset{i}{\sum }\frac{WFP_{i,t}-WFP_{i,0}}{lnWFP_{i,t}-lnWFP_{i,0}}ln\frac{P_{i,t}}{P_{i,0}}$$

### 2.5. Tapio Decoupling Elasticity Model

Tapio decoupling model was established by Tapio with the elasticity coefficient method [28]. According to the three critical values of 0, 0.8, and 1.2, Tapio decoupling state is divided into eight decoupling states (shown in Table 3). Specifically, strong decoupling is the ideal decoupling state because of the decline in resource consumption or environmental pressure with economic growth, while strong negative decoupling is the worst case scenario, reflecting that resource use continues to increase during the economic downturn. Generally, the water footprint should be on the decrease with the economic downturn. If the worst scenario really happens, it may be an artifact or caused by an economic crisis.

Tapio decoupling model comprehensively considers the total change and relative quantity change, and effectively avoids the limitations of base period selection, which exists in the traditional decoupling model, making relevant decoupling analysis more objective and accurate. In the decoupling study of resource consumption and economic growth, Tapio decoupling elasticity coefficient is defined as the ratio of the change rate of resource consumption or environmental pressure to the change rate of economic growth over a specific period. The calculation formula is shown below:

(14) $$X=\frac{EP_{t_{1}}-EP_{t_{0}}}{EP_{t_{0}}}/\frac{DP_{t_{1}}-DP_{t_{0}}}{DP_{t_{0}}}$$

In formula (14), *X* denotes decoupling elasticity, *EP* refers to environmental pressure, DP indicates the economic driving indicator, and $t_{0}$ and $t_{1}$ represent base period and current period, respectively. In this study, the environmental pressure (EP) is expressed by water footprint and its decomposition effect, and the economic driving force index (DP) is expressed by GDP [31,50].

## 3. Results

### 3.1. Measurement of Water Footprint

According to the above relevant formulas of water footprint, the results are shown in Table 4 and Table 5, and the proportions of water footprint composition indicators in BTHR are shown in Table 6.

Obviously, the total water footprint of BTHR increased gradually from 126.095 billion m^3^ in 2004 to 142.459 billion m^3^ in 2016, while the annual per capita water footprint in this region was generally on the decline, which was much lower than the national average over the same period [51].

**Figure 3:**
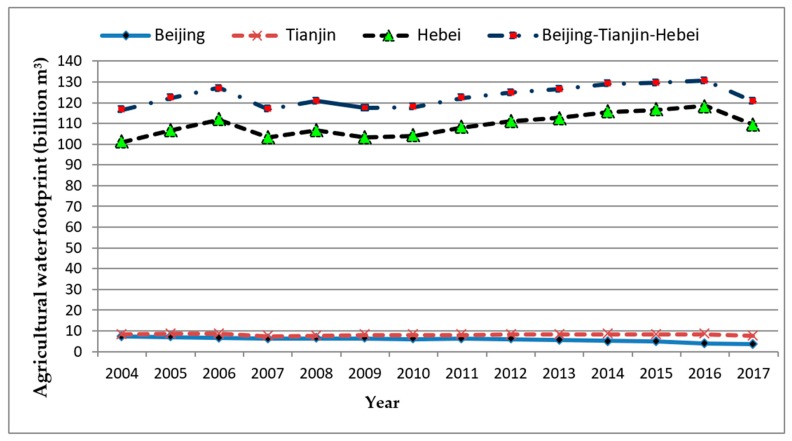
The change of agricultural water footprint in Beijing–Tianjin–Hebei region (BTHR) (2004–2017).

As shown in Table 6, the annual proportion of the agricultural water footprint in BTHR from 2004 to 2017 was over 90%. Furthermore, the agricultural water footprint of Hebei province exceeded 100 billion m^3^, accounting for more than 80% of the whole region due to larger land area and agricultural productions (Figure 3). Therefore, to reduce the water footprint, the main task of BTHR is to improve the agricultural water efficiency, and Hebei province needs to take more responsibilities in this process. As for the industrial water footprint of BTHR, it decreased year by year and accounted for 2%–3% of the total water footprint, indicating that there was high water utilization efficiency in the industrial production process in the region. Overall, the water footprint of residents showed a slow-growth trend from 2004 to 2017. The annual proportion of residential water footprint has increased slightly and remains at a low level of 3%–4%. With rapid economic growth in BTHR, local residents have more economic incomes to increase their demand for available fresh water. Meanwhile, as the South-to-North Water Diversion Project was launched in 2013, the water demand of people in BTHR has been met to a greater extent. However, due to the high cost of water and the strong awareness of water conservation, the water consumption of local residents did not increase rapidly [52]. In terms of ecological environmental water footprint, due to the strengthening awareness of ecological environmental protection, the water consumption for ecological environmental protection in BTHR has increased yearly from 2004 to 2017, and its annual proportion has increased by 6.9 times from the minimum value of 0.28% in 2004 to the maximum value of 1.96% in 2017. Obviously, this ratio still remains at a relatively low level. For the sake of ecological sustainable development, the amount of water resources used for ecological environmental protection should be appropriately increased in the future.

As for the virtual water trade, the virtual water import and the virtual water export are both decreasing in BTHR from 2004 to 2017 on the whole. However, the virtual water import is always larger than the virtual water export, which is closely related to the import of more water-intensive products [53]. 

Due to lack of water resources, the annual available water resources in BTHR, excluding the ecological environment demand, are about 1/60 of the average in the middle reaches of the Yangtze River [31] and 1/30 of the regional water footprint. Table 4 and Table 5 show that the water scarcity remains at a high level (1000–3000%), indicating a serious water crisis facing BTHR. In terms of water supply sources, the water resources self-sufficiency was higher than 94% from 2004 to 2017. Considering the huge gap between water demand and available water supply in China’s northern regions such as BTHR, the State Council of China formally approved the overall plan for the South-to-North Water Diversion Project in December 2002, aiming to solve the problem of uneven distribution of water resources across the country through external water diversion.

As mentioned above, water footprint only measures the actual utilization of water resource, while water footprint intensity describes the amount of water footprint consumed per unit of GDP, which reflects the water utilization efficiency better [54]. As shown in Table 5, the water footprint intensity of BTRH continued to decrease from 2004 to 2014, which decreased from the highest value of 0.09 m^3^/CNY in 2004 to its lowest value of 0.03 m^3^/CNY in 2014, indicating that the water utilization efficiency in BTHR has been on the rise. Compared with previous studies, it can be found out that the water footprint intensity in BTHR is much lower than that of most provinces and municipalities in China [40,54].

### 3.2. Analysis of Driving Factors of Water Footprint

**Figure 4:**
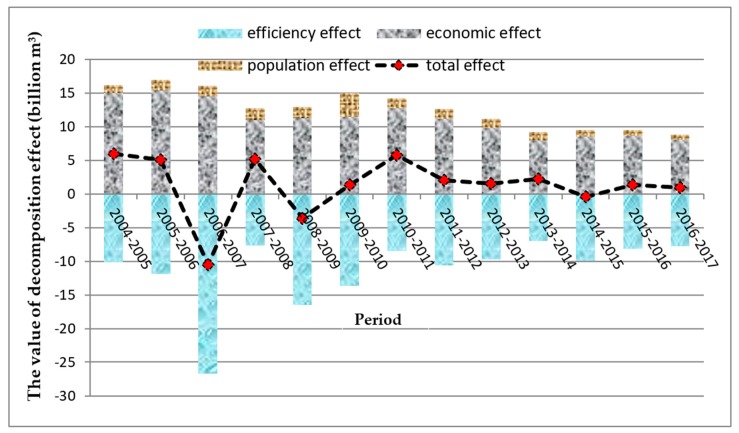
The change trend of water footprint’s (WF) logarithmic mean divisia index (LMDI) decomposition effect.

According to Equations (9)–(13), the change of water footprint can be decomposed into efficiency effect, economic effect, and population effect using LMDI model. The above calculation results are shown in Table 7 and Figure 4. In Table 7, the total effect (∆W) indicating the annual water footprint of BTHR increases by 17.168 billion m^3^ from 2004 to 2017. Meanwhile, the annual water footprint in this area has increased in most years, and its growth rate generally shows a downward trend. By contrast, the annual water footprint only decreased in 2006–2007, 2008–2009, and 2014–2015, with the largest decrease being in 2006–2007, which was 10.537 billion m^3^. As for the decomposition effects, the value of the efficiency effect is always negative and shows a trend of fluctuating decline. Thus, water utilization efficiency is the decisive factor of water footprint reduction. From 2004 to 2017, the efficiency effect reduced the water footprint of BTHR by 148.048 billion m^3^, which contributed the most to the reduction of the water footprint in 2006–2007, reaching 26.658 billion m^3^, while the contribution was only 6.993 billion m^3^ in 2013–2014, reaching the minimum value. 

Moreover, the economic effect and the population effect are always positive, and the economic effect is much larger than the population effect. The above results show that the improvement of economic development level and the expansion of population size will increase the regional water demand. However, economic development has a greater demand for water resources than population growth, which means it plays a more significant role in promoting the increase in water footprint. Thus, the economic effect is the main factor for the increase in water footprint, while the population effect has a minor effect on promoting the increase in water footprint in this region.

Through the LMDI method, the water footprint is decomposed from the provincial and municipal levels, and the results are shown in Table 8. The changes of water footprint in Beijing, Tianjin, and Hebei are mainly affected by efficiency effect and economic effect. Similar to the decomposition results above (Table 7), efficiency effect and economic effect are the main factors for the reduction and increase in water footprint in the three provinces/municipalities, respectively.

As for the total effect, the water footprint of the three provinces/municipalities showed an overall increasing trend from 2004 to 2017, among which the water footprints of Beijing and Hebei increased by 8.426 billion m^3^ and 7.035 billion m^3^, respectively, while Tianjin’s water footprint only increased by 1.708 billion m^3^. With larger increases in water footprint during the research period, Beijing and Hebei have a greater impact on the overall water footprint in BTHR. It is worth noting that the water footprint reduction of Hebei province reached the maximum of 11.344 billion m^3^ in 2016–2017, while the increase of Beijing and Tianjin reached the maximum of 11.156 billion m^3^ and 1.179 billion m^3^, respectively, in the same year. In particular, the efficiency effect becomes the main factor for the increase in water footprint in Beijing and Tianjin, which increased the water footprint of the two municipalities by 10.245 billion m^3^ and 804 million m^3^, respectively. In accordance with the above results, it can be seen that the water utilization efficiency of Beijing and Tianjin decreased in 2016–2017, among which Beijing decreased significantly. It reflects that Beijing and Tianjin consume more water to produce per unit of GDP in 2016–2017. This is because the two municipalities had more virtual water net imports in that year (shown in Table A1 and Table A2), and the water resource demand could be relatively satisfied, leading to the increase of water consumption for producing per unit of GDP.

Therefore, to reduce the water footprint in this region, more efforts should be made to develop water-saving technologies, especially more advanced agricultural irrigation technology, to improve the water utilization efficiency. Meanwhile, Beijing and Hebei should take greater responsibilities in reducing the water footprint.

### 3.3. Decoupling Analysis of Water Footprint and Economic Growth

**Figure 5:**
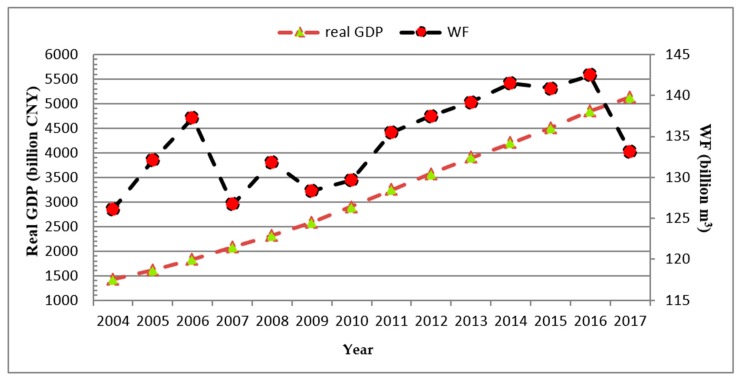
The change trend of water footprint and China’s real gross domestic product (GDP) in Beijing–Tianjin–Hebei region (BTHR) (2004–2017).

Figure 5 shows the changing trend of real GDP and water footprint in BTHR from 2004 to 2017. Clearly, the real GDP grows steadily and the water footprint fluctuates significantly. In addition, the growth rate of water footprint is lower than the economic growth rate in most years, indicating that the water utilization efficiency has improved in most years. According to the criteria of Tapio decoupling classification in Table 3, strong decoupling is the ideal state. At this time, water footprint decreases with the growth of economy, which reflects the improvement of water utilization efficiency. Similarly, weak decoupling represents that the economy and water footprint increase simultaneously, but the economic growth rate is larger than the water footprint, representing a huge progress in water utilization efficiency. Nevertheless, strong negative decoupling indicates the water footprint is on the rise during the economic downturn, which denotes the worst-case scenario.

Table 9 shows the decoupling state between water footprint and economic growth, and the driving factors of water footprint and economic growth in BTHR from 2004 to 2017. Since the economic effect and GDP growth are both economic indicators, there is no decoupling between them. The decoupling state between water footprint and GDP is manifested as weak decoupling in most years, which indicates that the water footprint in BTHR has experienced a decrease in some years while the GDP grows. Even in the growth years, the growth rate of water footprint is still lower than that of the GDP, making the water utilization efficiency rise during this period. Moreover, the water footprint intensity and GDP always show a strong decoupling state, depicting that the water footprint intensity in BTHR is decreasing with economic growth, so the water utilization efficiency is improving. Additionally, the population size and GDP always show an expansive coupling state, with the value of decoupling elasticity infinitely close to one, indicating that the annual growth rate of population size is basically the same as that of GDP.

## 4. Discussion

This study assessed the water utilization in BTHR based on water footprint method. Furthermore, we identified the driving factors affecting water footprint change and explored the decoupling relationship between water footprint and economic growth, and between the decomposition factors of water footprint and economic growth in BTHR combined with LMDI and Tapio methods. 

The results show that total water footprint of BTHR increased gradually from 126.095 billion m^3^ in 2004 to 142.459 billion m^3^ in 2016 (Table 4). Due to rapid economic development, BTHR, especially Beijing, has greater water demand, which has been satisfied to a large extent by transferring water through the South-to-North Water Diversion Project [55]. Meanwhile, the annual per capita water footprint in this region is generally declining, which is much lower than the national average over the same period (Table 5) [8,55], reflecting that there is relative higher water use efficiency mitigating the severe water crisis. It is the relatively advanced water-saving technology and strong residential awareness of water conservation in BTHR that counts. Compared with other provincial areas, BTHR, with lower agricultural proportion, should have consumed less water resources for agricultural production. However, the agricultural footprint in the BTHR accounts for over 90% of the total water footprint (shown in Table 6). Generally, the agricultural footprint is mainly affected by the irrigation technology [56], and the irrigation technology can only be improved through a long time. That is why BTHR’s agricultural footprint still accounts for a large proportion from 2004 to 2017.

Additionally, the change of water footprint can be attributed to efficiency effect, economic effect, and population effect. Specifically, the economic effect is the main driving factor for the increase of water footprint and population effect has exerted small influence on water footprint growth. Thus, it reflects economic growth requires higher water demand than population growth in the BTHR from 2004 to 2017. If the economic growth in the BTHR is mainly generated by the finance sector, the population effect may play a more important role in water footprint change. By contrast, the water utilization efficiency, reflecting the water consumption of per unit of GDP, is the decisive factor of water footprint reduction, which is closely related to residents’ awareness of water conservation [57,58,59] and water-saving technology, especially the irrigation efficiency. Although the mechanized irrigation plays an important role to improve agricultural production and reduce labor force, it also results in the squander of water resources due to inefficient irrigation. Since the agricultural production needs most available water, relevant water-saving irrigation technologies should be improved timely so as to play a pivotal role in reducing the consumption.

In terms of the results of the Tapio decoupling analysis, water footprint and GDP growth are in strong decoupling or weak decoupling, indicating that water footprint decreases or increase slower when the GDP continues to grow. Although water is a necessity for economic growth, it can be substituted to some degree. For instance, with wind, solar energy and other kinds of renewable energies further applied to generate electricity in China, hydropower dependence has been relieved to some extent. Thus, water resources have been less used or increased in electric power industry, while the economic development promoted by electric power keeps increasing. Meanwhile, the decoupling status between water footprint intensity and economic growth remains strong decoupling. In such an ideal status, water footprint intensity keeps decreasing as the economy grows. As a region highly dependent on transferred water from China’s southern provinces, BTHR has made tremendous efforts to improve water use efficiency while developing socio-economy. As a result, the water footprint intensity remains in a downward trend. In this research, water footprint intensity can be regarded as the driving factor of strong decoupling status between water footprint and economic growth. In general, the above decoupling states indicate that water utilization efficiency continues to improve with the development of economy [40]. Thus, BTHR has realized coordinated development between water consumption and economic growth to some extent.

Three feasible policy interventions could promote the coordinated development of water resource and economic growth. Firstly, policy makers should strongly support the research and development of water-saving technologies while developing local economy, especially for agricultural mechanized irrigation, so as to improve the water utilization efficiency [56,60]. Furthermore, more efforts should be made to adjust regional industrial structure. Secondly, local government departments should implement the reasonable tiered water price according to the monthly water consumption on the premise of fully considering the total amount of water resources and the level of economic development in different regions [52,57,58,59], so as to raise residents’ awareness of water conservation. Thirdly, while ensuring the export of domestic products, the import of water-intensive products should be appropriately increased to alleviate the pressure of regional water shortage in certain years [53,61,62].

## 5. Conclusions

Based on the water footprint theory, this paper calculated the water footprint and evaluated the utilization of water resources in BTHR from 2004 to 2017. Then, the LMDI method was used to decompose the driving factors affecting the change of water footprint. The dynamic change of water footprint was analyzed from the aspects of the total effect, efficiency effect, economic effect, and population effect. Finally, the Tapio decoupling model analyzed the decoupling states between water footprint and economic growth, and between the driving factors of water footprint and economic development. The following main conclusions can be drawn:

1. BTHR is suffering from a more serious water scarcity compared with the national average. Meanwhile, its water footprint is slowly increasing year by year, and the agricultural water footprint accounts for most of it. Additionally, the water utilization efficiency keeps improving, indicating less water is used to produce per unit of GDP, while the agricultural efficiency, mainly driven by water-saving irrigation technology, remains low level in the short term.
2. The change of water footprint can be decomposed into efficiency effect, economic effect, and population effect. Specifically, the economic effect is the main driving factor for the increase in water footprint. On the contrary, population effect has small influence on the increase of water footprint, while water utilization efficiency proves to be the decisive factor for the decrease in water footprint.
3. Water footprint and economic growth are in strong decoupling or weak decoupling, while the decoupling status between water footprint intensity and economic growth remains strong decoupling. Moreover, the decoupling status between population size and economic growth remains expansive coupling. Above decoupling states indicate that water utilization efficiency is improving.

Although this research has provided useful insights for water resources management decisions and policy implementations based on water footprint theory, two potential and important extensions could be made by further studies. (1) It is worthwhile to predict the dynamic change of the decoupling states between water footprint and economic growth. (2) The key factors affecting the coordinated development of water-economy nexus should be further explored.

## Figures and Tables

**Table 1:** Virtual water content per unit of crop products and livestock products (m^3^/kg).

Product	Virtual Water Content
Grain	1.13
Cotton	4.4
Oil plants	3.967
Vegetables	0.1
Fruit	0.82
Pork	2.21
Beef	12.56
Mutton	5.202
Poultry	3.652
Dairy	1.9
Eggs	3.55
Freshwater aquatic products	5

**Table 2:** Water footprint evaluation index.

Index Meaning	Formulas
Per capita water footprint (*PWFP*) represents the per capita consumption of water resource. The larger this index is, the more per capita water consumption is. (*P* refers to the population)	Equation (4)
Water import dependency (*WD*) is defined as the ratio of external water footprint and total water footprint. The larger this index is, the more virtual water import is.	Equation (5)
Water self-sufficiency (*WSS*) is represented as the ratio of internal water footprint to total water footprint. The larger this index is, the more internal water resources are used.	Equation (6)
Water scarcity (*WS*) measures the degree of regional water shortage. The higher this index is, the more serious the local water shortage is. (*WA* refers to the available water resources)	Equation (7)
Water footprint intensity (*WFI*) refers to the amount of regional water resources consumed per unit of GDP. The larger this index is, the lower the water utilization efficiency is.	Equation (8)

**Table 3:** The criteria for Tapio decoupling elasticity model.

Decoupling Type	∆*EP*	∆*DP*	*X*	Decoupling State
Negative decoupling	>0	>0	(1.2,+∞)	Expansive negative decoupling
	>0	<0	(−∞,0)	Strong negative decoupling
	<0	<0	(0,0.8)	Weak negative decoupling
Decoupling	>0	>0	(0,0.8)	Weak decoupling
	<0	>0	(−∞,0)	Strong decoupling
	<0	<0	(1.2,+∞)	Recessive decoupling
Coupling	>0	>0	(0.8,1.2)	Expansive coupling
	<0	<0	(0.8,1.2)	Recessive coupling

**Table 4:** The water footprint composition index in Beijing–Tianjin–Hebei region (BTHR) (2004–2017) (billion m^3^).

Year	Agricultural Water Footprint	Industrial Water Footprint	Residential Water Footprint	Ecological Water Footprint	Virtual Water Import	Virtual Water Export	Total Water Footprint	Internal Water Footprint	External Water Footprint
2004	116.494	3.791	3.902	0.352	5.538	3.982	126.095	120.557	5.538
2005	122.246	3.697	4.215	0.377	6.009	4.429	132.116	126.107	6.009
2006	127.236	3.685	4.309	0.327	6.203	4.530	137.231	131.028	6.203
2007	116.873	3.492	4.333	0.526	6.125	4.653	126.696	120.571	6.125
2008	120.734	3.423	4.360	0.703	7.145	4.484	131.881	124.736	7.145
2009	117.595	3.326	4.381	0.739	5.129	2.808	128.363	123.234	5.129
2010	117.941	3.295	4.476	0.806	6.187	3.033	129.672	123.485	6.187
2011	122.313	3.570	4.780	0.920	6.823	2.927	135.479	128.656	6.823
2012	124.998	3.520	4.430	1.090	6.134	2.671	137.500	131.366	6.134
2013	126.442	3.572	4.508	1.147	6.074	2.546	139.195	133.121	6.074
2014	128.958	3.500	4.610	1.440	5.554	2.606	141.456	135.902	5.554
2015	129.485	3.160	4.680	1.830	4.074	2.349	140.879	136.805	4.074
2016	130.838	3.120	4.930	2.190	3.507	2.126	142.459	138.952	3.507
2017	120.673	2.930	5.140	2.610	3.884	2.131	133.106	129.222	3.884

**Table 5:** The water footprint evaluation index in Beijing–Tianjin–Hebei region (BTHR) (2004–2017).

Year	Per capita Water Footprint (m^3^/person)	Water Import Dependency (%)	Water Self-sufficiency (%)	Water Scarcity (%)	Water Footprint Intensity (m^3^/CNY)
2004	1352.08	4.39%	95.61%	1660.89	0.09
2005	1400.72	4.55%	95.45%	1961.34	0.08
2006	1433.37	4.52%	95.48%	2459.33	0.07
2007	1301.58	4.83%	95.17%	2044.81	0.06
2008	1327.30	5.42%	94.58%	1544.27	0.06
2009	1267.46	4.00%	96.00%	1799.84	0.05
2010	1240.29	4.77%	95.23%	1893.58	0.04
2011	1276.29	5.04%	94.96%	1698.58	0.04
2012	1276.70	4.46%	95.54%	1116.44	0.04
2013	1273.25	4.36%	95.64%	1614.41	0.04
2014	1278.11	3.93%	96.07%	2561.08	0.03
2015	1264.29	2.89%	97.11%	2016.02	0.03
2016	1269.73	2.46%	97.54%	1356.01	0.03
2017	1273.77	2.92%	97.08%	1977.69	0.03

**Table 6:** The ratio of water footprint composition index in Beijing–Tianjin–Hebei Region (BTHR) (2004–2017).

Year	Agricultural Water Footprint	Industrial Water Footprint	Residential Water Footprint	Ecological Water Footprint	Virtual Water Import	Virtual Water Export
2004	92.39%	3.01%	3.09%	0.28%	4.39%	3.16%
2005	92.53%	2.80%	3.19%	0.29%	4.55%	3.35%
2006	92.72%	2.69%	3.14%	0.24%	4.52%	3.30%
2007	92.25%	2.76%	3.42%	0.42%	4.83%	3.67%
2008	91.55%	2.60%	3.31%	0.53%	5.42%	3.40%
2009	91.61%	2.59%	3.41%	0.58%	4.00%	2.19%
2010	90.95%	2.54%	3.45%	0.62%	4.77%	2.34%
2011	90.28%	2.64%	3.53%	0.68%	5.04%	2.16%
2012	90.91%	2.56%	3.22%	0.79%	4.46%	1.94%
2013	90.84%	2.57%	3.24%	0.82%	4.36%	1.83%
2014	91.16%	2.47%	3.26%	1.02%	3.93%	1.84%
2015	91.91%	2.24%	3.32%	1.30%	2.89%	1.67%
2016	91.84%	2.19%	3.46%	1.54%	2.46%	1.49%
2017	90.66%	2.20%	3.86%	1.96%	2.92%	1.60%

**Table 7:** The logarithmic mean divisia index (LMDI) analysis of water footprint (WF) change in Beijing–Tianjin–Hebei region (BTHR) (billion m^3^).

Year	WF Change			
	Efficiency Effect	Economic Effect	Population Effect	Total Effect
2004–2005	−10.152	14.985	1.188	6.020
2005–2006	−11.848	15.428	1.535	5.115
2006–2007	−26.658	14.551	1.573	−10.534
2007–2008	−7.610	10.982	1.813	5.184
2008–2009	−16.510	11.266	1.656	−3.588
2009–2010	−13.686	11.504	3.562	1.380
2010–2011	−8.449	12.789	1.466	5.806
2011–2012	−10.629	11.209	1.442	2.022
2012–2013	−9.673	9.840	1.372	1.539
2013–2014	−6.993	7.909	1.314	2.230
2014–2015	−9.906	8.564	0.953	−0.39
2015–2016	−8.129	8.681	0.842	1.394
2016–2017	−7.804	8.067	0.728	0.991
Sum	−148.048	145.772	19.444	17.168

**Table 8:** The logarithmic mean divisia index (LMDI) analysis of water footprint (WF) change in Beijing, Tianjin, and Hebei (billion m^3^).

Period	Beijing				Tianjin				Hebei			
	Efficiency Effect	Economic Effect	Population Effect	Total Effect	Efficiency Effect	Economic Effect	Population Effect	Total Effect	Efficiency Effect	Economic Effect	Population Effect	Total Effect
2004–2005	−1.399	0.966	0.350	−0.083	−1.054	1.128	0.175	0.248	−7.699	12.891	0.663	5.855
2005–2006	−1.509	0.940	0.470	−0.099	−1.323	1.011	0.290	−0.021	−9.016	13.477	0.775	5.235
2006–2007	−1.938	0.902	0.522	−0.514	−2.610	0.935	0.325	−1.349	−22.110	12.714	0.726	−8.671
2007–2008	0.123	0.363	0.645	1.132	−0.998	0.835	0.447	0.284	−6.735	9.784	0.721	3.769
2008–2009	−2.295	0.562	0.573	−1.160	−0.846	0.961	0.380	0.495	−13.368	9.743	0.703	−2.922
2009–2010	−0.486	0.510	0.610	0.635	−1.258	0.952	0.513	0.207	−11.942	10.042	2.438	0.538
2010–2011	−0.124	0.599	0.348	0.823	−1.206	1.025	0.394	0.213	−7.119	11.166	0.723	4.770
2011–2012	−1.258	0.617	0.303	−0.338	−1.166	0.828	0.397	0.060	−8.205	9.764	0.741	2.300
2012–2013	−1.299	0.628	0.265	−0.407	−1.101	0.732	0.389	0.021	−7.274	8.480	0.718	1.925
2013–2014	−1.491	0.610	0.199	−0.682	−0.718	0.628	0.290	0.200	−4.783	6.671	0.825	2.713
2014–2015	−1.730	0.616	0.093	−1.021	−0.826	0.677	0.191	0.041	−7.349	7.271	0.668	0.590
2015–2016	−1.646	0.623	0.009	−1.015	−0.727	0.762	0.095	0.130	−5.756	7.296	0.738	2.278
2016–2017	10.245	0.925	−0.015	11.156	0.804	0.408	−0.034	1.179	−18.854	6.733	0.776	−11.344
Sum	−4.807	8.860	4.374	8.426	−13.028	10.881	3.854	1.708	−130.212	126.030	11.216	7.035

**Table 9:** Decoupling status of water footprint (WF) and its driving factors and gross domestic product (GDP).

Year	Decoupling Elasticity of WFI (Water Footprint Intensity) and GDP	Decoupling Status	Decoupling Elasticity of PS (Population Size) and GDP	Decoupling Status	Decoupling Elasticity of WF (Water Footprint) and GDP	Decoupling Status
2004–2005	−0.56411	Strong decoupling	1.00042	Expansive coupling	0.36136	Weak decoupling
2005–2006	−0.62775	Strong decoupling	1.00105	Expansive coupling	0.287808	Weak decoupling
2006–2007	−1.3867	Strong decoupling	0.99701	Expansive coupling	−0.5726	Strong decoupling
2007–2008	−0.56787	Strong decoupling	0.99683	Expansive coupling	0.36919	Weak decoupling
2008–2009	−1.11147	Strong decoupling	1.00031	Expansive coupling	−0.23834	Strong decoupling
2009–2010	−0.81198	Strong decoupling	0.99955	Expansive coupling	0.083724	Weak decoupling
2010–2011	−0.55138	Strong decoupling	0.99878	Expansive coupling	0.384389	Weak decoupling
2011–2012	−0.77425	Strong decoupling	0.99778	Expansive coupling	0.147319	Weak decoupling
2012–2013	−0.80404	Strong decoupling	1.00118	Expansive coupling	0.122505	Weak decoupling
2013–2014	−0.73278	Strong decoupling	1.00276	Expansive coupling	0.211706	Weak decoupling
2014–2015	−0.96484	Strong decoupling	0.99775	Expansive coupling	−0.03693	Strong decoupling
2015–2016	−0.80668	Strong decoupling	0.99762	Expansive coupling	0.133597	Weak decoupling
2016–2017	−0.83219	Strong decoupling	1.00138	Expansive coupling	0.119194	Weak decoupling

## Appendix A

**Table A1:** Water footprint composition index in Beijing from 2004 to 2017 (billion m^3^).

Year	Agricultural Water Footprint	Industrial Water Footprint	Residential Water Footprint	Ecological Water Footprint	Virtual Water Import	Virtual Water Export	Total Water Footprint
2004	7.158	0.766	1.291	0.100	3.496	0.971	11.840
2005	6.960	0.680	1.393	0.110	3.880	1.265	11.758
2006	6.631	0.620	1.443	0.162	4.098	1.295	11.659
2007	6.213	0.575	1.460	0.272	3.975	1.350	11.145
2008	6.291	0.520	1.533	0.320	4.936	1.324	12.276
2009	6.345	0.520	1.533	0.360	3.324	0.966	11.116
2010	6.074	0.506	1.530	0.397	4.188	0.945	11.751
2011	6.101	0.500	1.630	0.450	4.739	0.846	12.573
2012	5.927	0.490	1.600	0.570	4.403	0.754	12.236
2013	5.590	0.512	1.625	0.592	4.239	0.729	11.829
2014	5.075	0.510	1.700	0.720	3.815	0.673	11.147
2015	4.784	0.380	1.750	1.040	2.737	0.565	10.126
2016	4.051	0.380	1.780	1.110	2.311	0.522	9.109
2017	3.490	0.350	1.830	1.270	2.525	0.557	8.908

**Table A2:** Water footprint composition index in Tianjin from 2004 to 2017 (billion m^3^).

Year	Agricultural Water Footprint	Industrial Water Footprint	Residential Water Footprint	Ecological Water Footprint	Virtual Water Import	Virtual Water Export	Total Water Footprint
2004	8.349	0.507	0.453	0.048	1.242	1.225	9.374
2005	8.743	0.451	0.454	0.045	1.271	1.341	9.622
2006	8.752	0.443	0.461	0.049	1.288	1.393	9.601
2007	7.465	0.420	0.482	0.051	1.160	1.326	8.252
2008	7.697	0.381	0.488	0.065	0.929	1.024	8.536
2009	7.893	0.435	0.509	0.109	0.721	0.637	9.030
2010	7.965	0.483	0.548	0.122	0.744	0.624	9.237
2011	8.111	0.500	0.540	0.110	0.775	0.586	9.451
2012	8.146	0.510	0.500	0.140	0.761	0.546	9.511
2013	8.240	0.537	0.505	0.090	0.823	0.507	9.688
2014	8.397	0.540	0.500	0.210	0.765	0.495	9.918
2015	8.346	0.530	0.490	0.290	0.611	0.495	9.772
2016	8.428	0.550	0.560	0.410	0.590	0.447	10.090
2017	7.658	0.550	0.610	0.520	0.694	0.444	9.588

**Table A3:** Water footprint composition index in Hebei from 2004 to 2017 (billion m^3^).

Year	Agricultural Water Footprint	Industrial Water Footprint	Residential Water Footprint	Ecological Water Footprint	Virtual Water Import	Virtual Water Export	Total Water Footprint
2004	100.986	2.518	2.158	0.204	0.801	1.786	104.881
2005	106.544	2.566	2.368	0.222	0.858	1.822	110.735
2006	111.853	2.622	2.405	0.116	0.817	1.843	115.971
2007	103.196	2.497	2.391	0.203	0.990	1.977	107.300
2008	106.747	2.522	2.339	0.318	1.279	2.136	111.069
2009	103.357	2.371	2.339	0.270	1.084	1.206	108.216
2010	103.901	2.306	2.398	0.287	1.255	1.464	108.684
2011	108.101	2.570	2.610	0.360	1.309	1.495	113.454
2012	110.925	2.520	2.330	0.380	0.970	1.371	115.754
2013	112.612	2.523	2.377	0.465	1.012	1.310	117.679
2014	115.486	2.450	2.410	0.510	0.973	1.438	120.391
2015	116.355	2.250	2.440	0.500	0.725	1.289	120.981
2016	118.359	2.190	2.590	0.670	0.607	1.157	123.260
2017	109.525	2.030	2.700	0.820	0.665	1.130	114.610
